# Sepsis subphenotypes: bridging the gaps in sepsis treatment strategies

**DOI:** 10.3389/fimmu.2025.1546474

**Published:** 2025-02-06

**Authors:** Xue Zhang, Wei Zhang, Huan Zhang, Xuelian Liao

**Affiliations:** ^1^ Department of Critical Care Medicine, West China Hospital, Sichuan University, Chengdu, Sichuan, China; ^2^ Institute of Critical Care Medicine, State Key Laboratory of Biotherapy and Cancer Center, West China Hospital, Sichuan University, Chengdu, Sichuan, China; ^3^ Department of Critical Care Medicine, West China Tianfu Hospital, Sichuan University, Chengdu, Sichuan, China

**Keywords:** sepsis heterogeneity, phenotyping, personalized therapy, phenotype-driven treatment, biomarkers, therapeutic target identification

## Abstract

Sepsis, a heterogeneous illness produced by a dysregulated host response to infection, remains a severe mortality risk. Recent discoveries in sepsis research have stressed phenotyping as a feasible strategy for tackling heterogeneity and enhancing therapy precision. Sepsis phenotyping has moved from traditional stratifications based on severity and prognosis to dynamic, phenotype-driven therapeutic options. This review covers recent progress in connecting sepsis subgroups to personalized treatments, with a focus on phenotype-based therapeutic predictions and decision-support systems. Despite ongoing challenges, such as standardizing phenotyping frameworks and incorporating findings into clinical practice, this topic has enormous promise. By investigating phenotypic variation in therapy responses, we hope to uncover new biomarkers and phenotype-driven therapeutic solutions, laying the groundwork for more effective therapies and, ultimately improving patient outcomes.

## 1 Introduction

Sepsis is a primary cause of mortality among critically sick patients worldwide, accounting for approximately 49 million cases and 11 million deaths each year, representing nearly 20% of all deaths worldwide (1–3). It results from an anomalous immunological response to infection that precipitates organ failure (1, 4). Sepsis exhibits high clinical heterogeneity, making the discovery of effective treatments exceedingly challenging (5). Despite advances in understanding the cellular and molecular causes of sepsis, its complicated pathophysiology remains a problem (6). This heterogeneity arises from diverse pathogens, infection sites, and host immune responses that interact intricately to shape the clinical presentation (7–11). The phenotypical variability in sepsis is reflected across various clinical manifestation. Hemodynamic alterations, including blood pressure, cardiac output, and systemic vascular resistance, are prevalent indicators of sepsis-induced circulatory dysfunction (12, 13). Coagulation diseases, including disseminated intravascular coagulation (DIC), platelet failure, and microthrombosis, intensify organ damage (14, 15). Neurological manifestations encompass sepsis-associated encephalopathy and delirium, reflecting both direct and indirect impacts on the central nervous system (16, 17). Metabolic instability, including hyperlactatemia and mitochondrial dysfunction, underscores the systemic nature of sepsis (18, 19). Respiratory issues, especially acute respiratory distress syndrome (ARDS) and hypoxemia, often predominate the clinical trajectory, however other systems, including renal and hepatic, exhibit varied responses based on the patient’s phenotype (13, 20–22).

Given this multifaceted heterogeneity, understanding how these clinical factors interplay to shape sepsis phenotypes is essential. Recent advancements in big data methodologies, artificial intelligence, and high-throughput multi-omics data, along with disease phenotyping, offer novel pathways for enhancing the comprehension of sepsis and formulating more personalized treatment strategies (23). Phenotypic approaches employ computational tools to analyze clinical, biomarker, and genetic data, aiming to discover more homogeneous subpopulations within sepsis patients (23, 24). This technology facilitates precise therapy delivery, guaranteeing that the appropriate patient receives the exact treatment at the designated time. In other fields, such as oncology, phenotypic methodologies have enhanced therapeutic accuracy (25, 26). Leveraging these advancements, sepsis treatment could be revolutionized, perhaps leading to the identification of new biomarkers and therapeutic targets that correspond to phenotypic differences in treatment response, thus enhancing patient outcomes.

**Figure 1:**
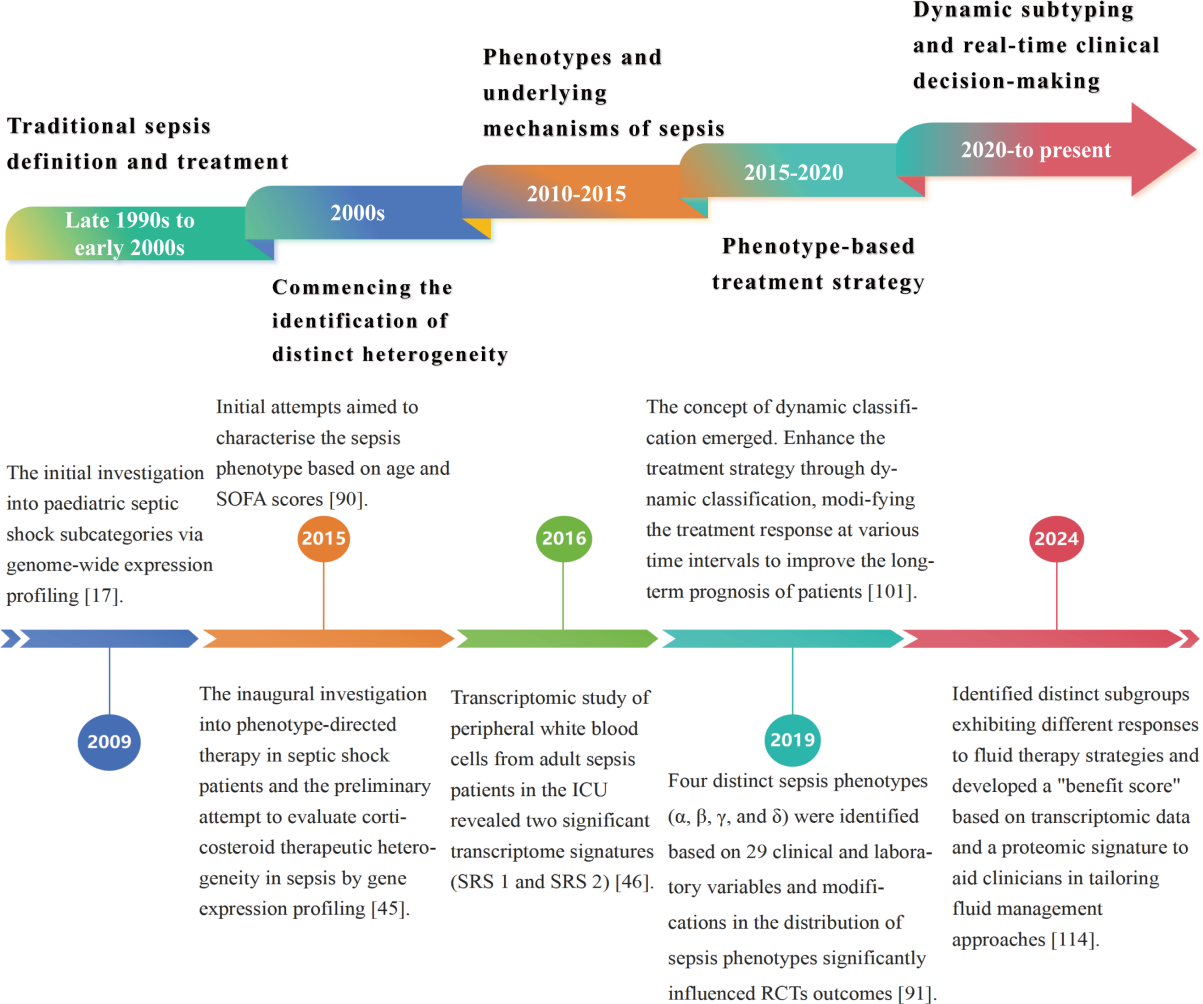
Chronology of the developmental history of sepsis classification. The evolution of sepsis phenotypic research from static categorization to dynamic classification has led to increasingly customized and precise treatment approaches for sepsis. Precise phenotypic classification and phenotype-based therapy strategies enhance understanding of sepsis heterogeneity, identify novel therapeutic targets, and optimize clinical treatment protocols, ultimately improving patient outcomes. Future research will focus on improving treatment outcomes through real-time monitoring and dynamic modifications to treatment protocols, aimed at addressing existing challenges in precision therapy.

This review examines current advancements in sepsis phenotyping (**Figure 1**), highlighting the potential of these methods to tackle sepsis heterogeneity and optimize treatment protocols. We aim to establish a foundation for the identification of novel biomarkers and targeted treatments based on differential responses among sepsis subtypes, ultimately enhancing patient outcomes.

## 2 The necessity of sepsis classification

**Figure 2:**
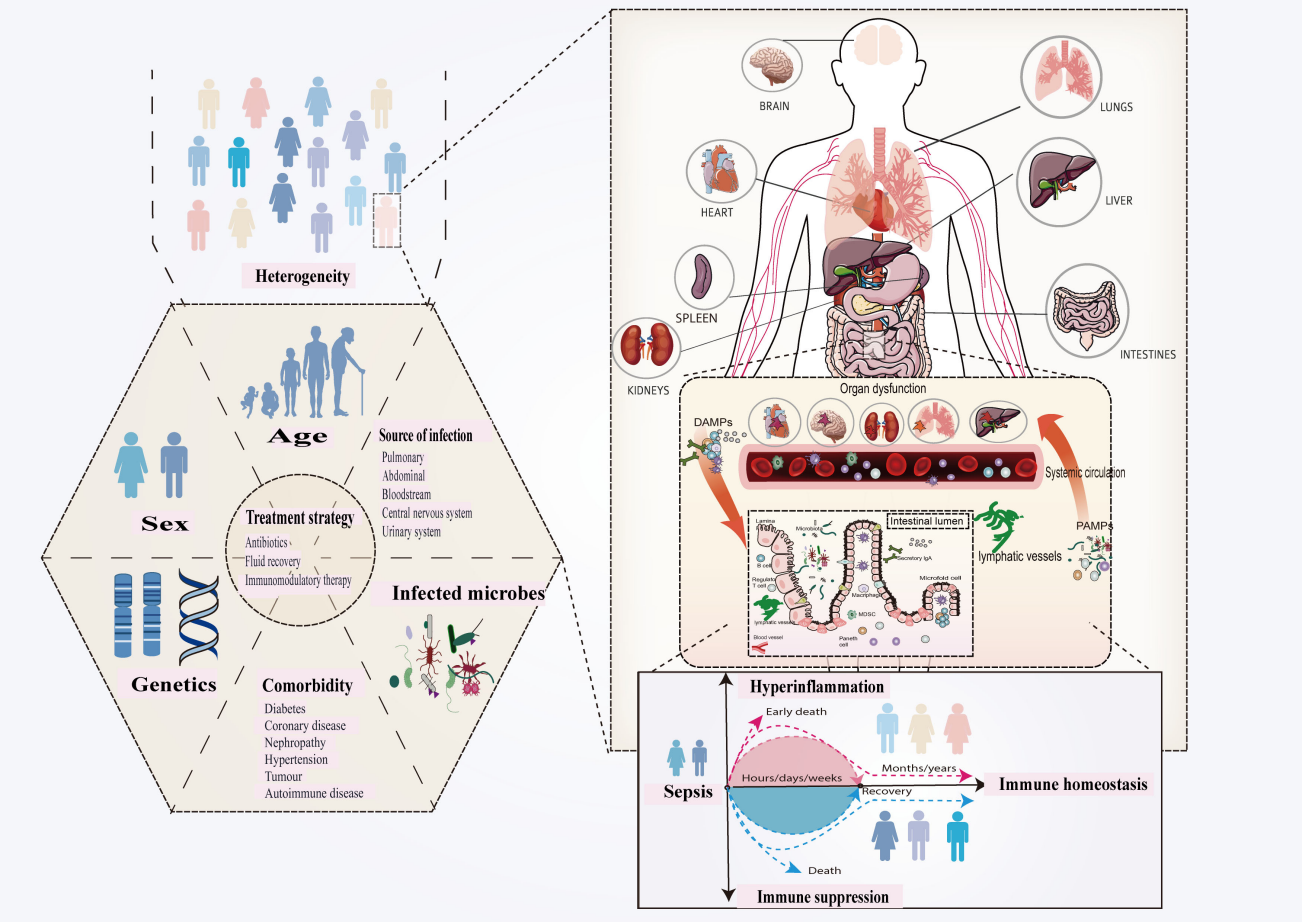
Sepsis is defined as a dysregulated host response to infection that leads to organ dysfunction. Innate immunity is inherently structured to swiftly respond to conserved molecules known as PAMPs and DAMPs, which are produced by infections and hosts, respectively. The complexities of pathogen-host interactions increase the variability of sepsis manifestations. The observed heterogeneity primarily results from pre-existing individual health conditions, variability in pathogenic bacteria, differences in infection sites, and variations in host immune responses. This variety underpins differences in clinical presentation, prognosis, and treatment outcomes, highlighting the importance of understanding sepsis diversity. Improving phenotype-based therapy techniques, identifying novel markers and targets through diverse therapeutic responses, and stratifying patients can all improve results and steer newer drugs. Pathogen-associated molecular patterns (PAMPs) and damage-associated molecular patterns (DAMPs).

Sepsis is a complex clinical disease defined by a variety of immunological responses, infection locations, and patient characteristics (7, 27). It is now regarded as a coexisting, dynamic proinflammatory and immunosuppressive route driven by pathogen virulence, host immunological regulation, and pharmaceutical interventions (27–30). Sepsis-induced immunological dysregulation, which includes genetic and metabolic changes to immune cells, causes organ failure and increases the chance of re-infection after pathogen clearance (31–36). Patient demographics, comorbidities, infection characteristics, and treatment timing all contribute to sepsis heterogeneity, which has an impact on clinical symptoms and treatment outcomes (**Figure 2**). Despite advances in sepsis research, a lack of specialized therapy options limits further success (36, 37).

### 2.1 Failures of randomized clinical trials in sepsis

In recent decades, extensive clinical trials designed to enhance sepsis treatment have predominantly failed to demonstrate significant clinical efficacy (38–46). The trials examined several medications, including hormone therapy, immunomodulators, antibiotics, and fluid resuscitation methods. Despite these efforts, various medications, such as recombinant activated protein C, have not reliably decreased mortality or enhanced organ function, with some associated with adverse side effects (38, 40, 46). Likewise, the reliance on therapies targeting individual pathways, such as anti-inflammatory cytokines like IL-1 and IL-6, fails to address the complex interplay of pro-inflammatory and anti-inflammatory responses in sepsis (41, 42). The primary reason for these failures lies in the assumption that sepsis is a homogenous condition, treatable with a “one-size-fits-all” approach. Research on antibiotics and fluid resuscitation has underscored the intricacies of sepsis management, revealing a lack of consensus regarding optimal treatment protocols (43–45, 47–49). Many RCTs lack robust stratification of patients by phenotype, leading to diluted efficacy signals and conflicting results. An example of this heterogeneity can be seen in patients with macrophage activation syndrome (MAS), a hyperinflammatory condition associated with sepsis. Studies have shown that these patients may benefit from IL-1 receptor antagonists, highlighting the importance of targeted therapies for specific subgroups (41). These insights underscore the need to move beyond conventional RCT designs and develop phenotype-driven approaches that tailor interventions to patient-specific profiles.

The failures of these trials highlight the importance of understanding sepsis heterogeneity at the molecular and clinical levels. Stratifying patients by genetic, molecular, and clinical phenotypes could enhance the precision of trials and allow the identification of subgroup-specific responses. And adaptive designs that allow modifications based on interim data can better accommodate the dynamic nature of sepsis and its heterogeneous population. Meanwhile, addressing the interplay of multiple pathways using combination therapies or network-driven strategies may prove more effective than single-target interventions. By addressing these issues, future trials can better align with the complexity of sepsis and pave the way for more effective treatments.

### 2.2 Deciphering sepsis heterogeneity: implications for phenotype-based therapies

Progress in genomic and transcriptomic technologies has revealed that sepsis has several subphenotypes, each associated with distinct clinical consequences. These findings highlight the promise of phenotype-driven therapies to surpass the limitations of traditional sepsis treatments. Initial research utilizing genetic analysis identified numerous sepsis subphenotypes exhibiting diverse clinical outcomes. An investigation of whole genome expression in whole blood RNA from 98 adolescent septic shock patients in 2009 uncovered numerous novel findings (24). This is the inaugural phenotypic investigation of septic shock patients, revealing three subphenotypes characterized by distinct immune response patterns and varying degrees of illness severity (24). Subsequent research corroborated these findings, indicating that gene expression profiling can delineate sepsis subgroups with varying treatment responses (50–52). In a 2015 study, researchers employed NanoString nCounter to quantify messenger RNA (mRNA) for 100 categorized genes, demonstrating gene expression mosaicism. Notwithstanding the enhancement, the model only identified subphenotypes A and B in the two cohorts (n=168 and n=132) (52). Subtype A activates glucocorticoid receptors to a lesser extent than subtype B (52). This prompted researchers to investigate adjuvant corticosteroid therapy and its prognostic implications. Adjuvant corticosteroids were administered to 52 (43%) of 120 patients with subphenotype A and 104 (58%) of 180 patients with subtype B. Corticosteroid therapy markedly decreased mortality in subtype A, but not in subtype B (52). This research is the first investigation of phenotype-directed therapy in septic shock patients by gene expression profiling to ascertain therapeutic heterogeneity in sepsis. Nonetheless, the administration of corticosteroids is non-randomized.

Furthermore, recent studies have shown demonstrated diversity in sepsis recovery (53–55). Research indicates that long-term outcomes differ, with numerous survivors facing persistent physical and psychological challenges (56–62). A 2021 study identified three distinct paths of depressive symptoms in septic shock survivors, highlighting the importance of personalized post- intensive care unit (ICU) care (59). Another study of clinical and biomarker data from 467 septic shock survivors identified two subgroups post-ICU discharge: Type A, characterized by low multi-organ dysfunction, and Type B, with a higher one-year mortality rate (34% vs. 16%) (55). Enhancing patient outcomes necessitates comprehending sepsis heterogeneity and developing phenotype-based therapeutics. The integration of epigenomics, transcriptomics, proteomics, metabolomics and cytomics with AI technologies is essential for advancing precision therapies in sepsis. This integration aids in identifying biomarkers and molecular signatures associated with sepsis subphenotypes, enhancing patient classification and treatment development. By integrating genetic and clinical data, researchers might enhance their comprehension of sepsis heterogeneity and develop stratified trial designs that consider subphenotype responses. This methodology establishes a robust framework for the identification of novel biomarkers and therapeutic targets, facilitating personalized treatments that enhance clinical outcomes and optimize therapeutic interventions.

## 3 Bridging sepsis subphenotypes to treatment strategies

**Figure 3:**
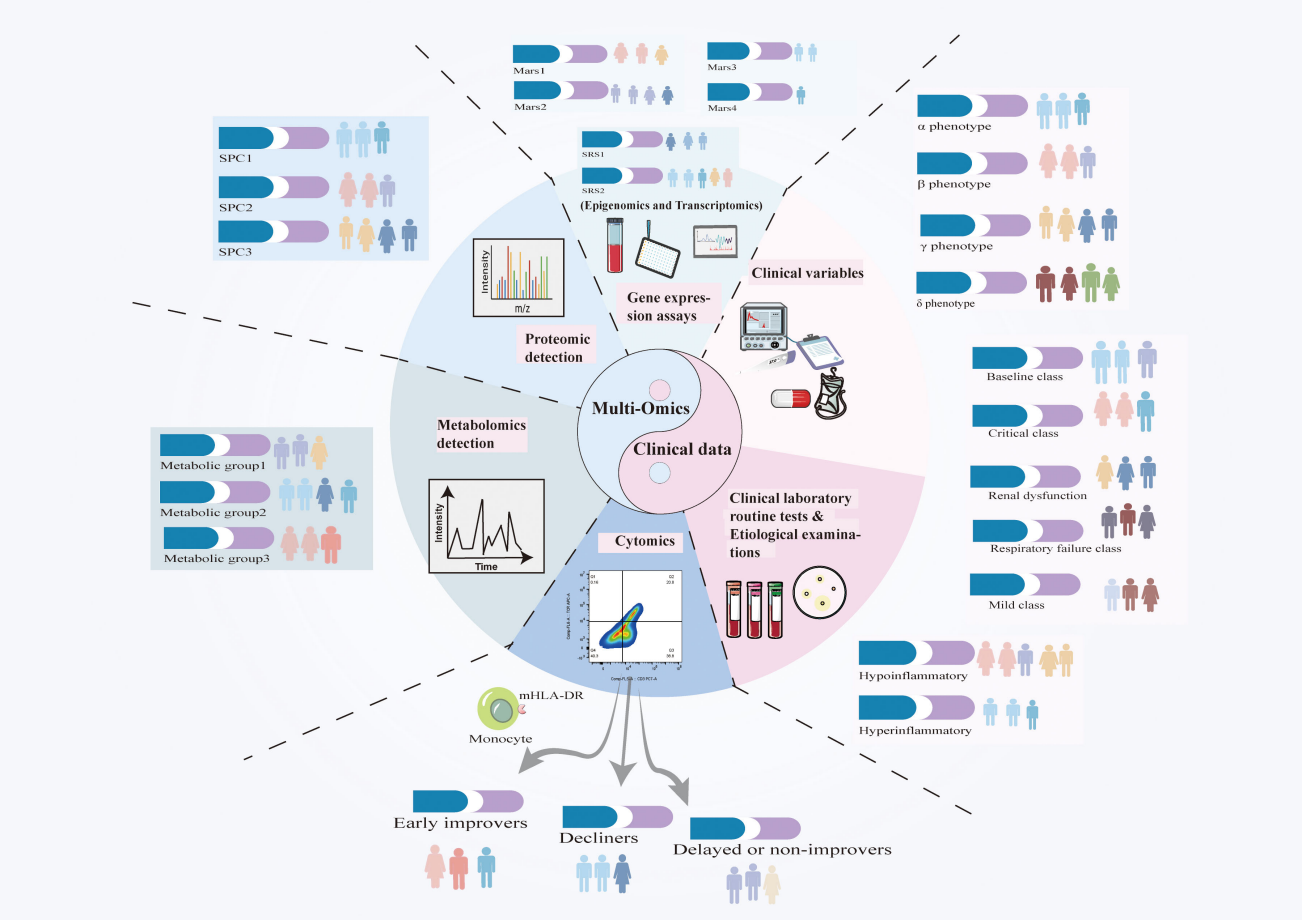
Advancements in big data and artificial intelligence (AI) have enhanced the integration of clinical, biomarker, and molecular data, facilitating a deeper understanding of sepsis heterogeneity. Although classifications of sepsis subtypes differ due to variations in study designs and methodologies, these technologies facilitate the identification of novel biomarkers and therapeutic targets. The integration of multi-omics data with artificial intelligence is essential for advancing precision medicine, refining patient stratification, and directing phenotype-based therapy. By integrating genetic and clinical data, researchers can enhance the comprehension of sepsis heterogeneity and formulate stratified trials, facilitating the development of personalized medications that optimize patient outcomes.

Sepsis exhibits significant variability and presents management challenges; thus, defining its subphenotypes is essential for improving treatment. Recently emerging multi-omics technologies, including epigenomics, transcriptomics, proteomics, metabolomics, and cytomics, have enabled the molecular classification of sepsis (**Figure 3**). When integrated with vital signs, biochemical markers, and evaluations of organ function, these technologies elucidate the pathophysiological underpinnings of sepsis. Epigenomic changes, such as DNA methylation and histone irregularities, influence immunological responses and disease outcomes. Transcriptomic and proteomic analyses reveal gene expression patterns and protein biomarkers associated with clinical variability in sepsis patients, facilitating the identification of biomarkers and therapeutic targets. Moreover, artificial intelligence (AI) and machine learning (ML) are extensively employed to analyze these high-dimensional datasets to identify novel disease patterns and predict patient outcomes (**Table 1**). K-means clustering and latent profile analysis have been employed to identify clinically significant subgroups, categorizing patients according to clinical and genomic data to facilitate more personalized therapeutic decisions (63–65). Supervised learning methods such as random forests and logistic regression can identify the onset of sepsis and enhance fluid resuscitation and antibiotic treatment (64, 66–70). AI/ML methodologies integrated with clinical data can design individualized therapy regimens that enhance clinical outcomes (71–77). Artificial Intelligence and Machine Learning exhibit potential (78, 79); yet, their incorporation into clinical practice necessitates thorough validation and external verification to ensure their dependability and relevance across diverse healthcare environments.

**Table 1:** Overview of the methods of machine learning and artificial intelligence.

Category	Method	Primary Application	Key Features	References
Unsupervised	K-means Clustering	Identifying subphenotypes	Simple, interpretable; sensitive to outliers	Huang et al. (64)
	Latent Profile Analysis	Revealing subphenotypes	Captures heterogeneity; requires large datasets	Liu et al. (65)
	Self-Organizing Maps	Clustering high-dimensional data	Effective visualization; computationally intensive	De Zuani et al. (74)
Supervised	Logistic Regression	Predicting onset, severity	Simple for binary outcomes; limited to linear relationships	Sun et al. (67)
	Random Forest	Predicting outcomes	Robust, non-linear; computationally expensive	Kijpaisalratana et al. (73)
	Decision Tree	Treatment guidance	Easy to interpret; prone to overfitting	Wang et al. (72)
	Support Vector Machines	Classifying subphenotypes	Effective for high-dimensional data; sensitive to parameter tuning	Turki et al. (76)
	Gradient Boosting/XGBoost	Predicting outcomes	High accuracy; computationally intensive	Berg et al. (70)
	Deep Learning	Time-series prediction	Effective for sequential data; requires large datasets	Lauritsen et al. (75)
	Artificial Neural Networks	Personalized treatment prediction	Processes high-dimensional data; requires large datasets	Loftus et al. (77)
	K-Nearest Neighbors	Predicting incidence	Intuitive; sensitive to irrelevant features	Michelson et al. (68)
	Ensemble Methods	Improving classification accuracy	Reduces overfitting; difficult to interpret	Wang et al. (72)
	Transfer Learning	Adapting models for data-scarce settings	Effective with pre-trained models; transfer not always perfect	Dieckhaus et al. (69)
Specialized	Latent Class Analysis	Classifying latent subgroups	Identifies hidden groups; sensitive to class assumptions	Cai et al. (63)
	Dynamic Treatment Regime	Personalized treatment strategies	Dynamic recommendations; complex computation	Ma et al. (152)
	Dynamic Time Warping	Aligning and comparing time-series data	Effective for sequence comparison; computationally expensive	Bhavani et al. (71)

### 3.1 Multi-omics-based studies

Sepsis is complex, thus knowing its molecular basis is crucial. Integrating Epigenomics, transcriptomics, proteomics, metabolomics, and cytomics data offers a comprehensive view of sepsis phenotyping (**Figure 3**). These methodologies delineate molecular subphenotypes, categorize patients, and formulate personalized treatment strategies. The following discussion addresses each omics technology and their potential synergy in the understanding and management of sepsis.

#### 3.1.1 Epigenomics

Epigenomics examines heritable modifications in gene expression that do not include alterations to the DNA sequence, including DNA methylation, histone modifications, and non-coding RNA. These epigenetic modifications significantly influence the immune response to sepsis. One study identified unique DNA methylation patterns in sepsis patients, correlating hypermethylation of immune regulatory genes with impaired immunological function (80). Another investigation identified histone modifications associated with sepsis-induced immune tolerance, offering insights into potentially reversible mechanisms (81). These findings underscore the epigenome’s pivotal role in defining molecular subtypes of sepsis and its potential therapeutic implications. Nonetheless, despite its promise, epigenomics possesses constraints. Epigenetic modifications can be affected by numerous environmental and temporal factors, complicating the establishment of definitive causality in sepsis. Moreover, the transient nature of many epigenetic modifications, coupled with the intricate relationships within the epigenome, complicates the ability to reach definitive findings. Epigenomics provides essential insights into the regulatory mechanisms of sepsis, sometimes complemented by transcriptomics, which captures real-time gene expression alterations and reveals more dynamic molecular endophenotypes of the condition.

#### 3.1.2 Transcriptomics

Transcriptomics focuses on gene expression profiling to classify molecular endophenotypes of sepsis, which reveal significant variations in treatment responses and outcomes. Cohort studies employing unsupervised clustering to assess blood leukocyte gene expression data typically discern molecular subphenotypes of sepsis (82, 83). Comprehending the diverse forms of sepsis and tailoring treatment to specific phenotypes can enhance patient outcomes. Multi-omics approaches, particularly transcriptomics, have provided significant insights into the immunological dysregulation and genetic markers that characterize sepsis subphenotypes (82–84).

Transcriptomics-based studies have identified several molecular subphenotypes associated with distinct immune responses in sepsis patients. For example, a transcriptomic analysis of peripheral white blood cells from 265 adult sepsis patients in the ICU with community-acquired pneumonia was published in 2016. This investigation identified two significant transcriptome signatures—SRS 1 and SRS 2—that are associated with a bad and favorable prognosis, respectively (82). These signatures were confirmed across numerous cohorts and contributed to the development of gene classifiers capable of accurately predicting sepsis subphenotypes (82, 85, 86). Importantly, SRS 1 was associated with immunosuppression, whereas SRS 2 was associated with a more active immune response (82). Moreover, dynamic changes in SRS subgroup membership were observed during ICU stays (85).

A significant issue in transcriptomics research has been the absence of standardized protocols, leading to variability among studies. Recent initiatives to enhance research methodologies and replicate results across cohorts have yielded more dependable and reproducible outputs. For example, a gene expression analysis utilizing whole blood mRNA microarray data from diverse infection sources, including pneumonia and peritonitis, identified similarities and differences in immune responses, contributing to the characterization of sepsis subphenotypes (Mars1-4) (83). The Mars1 endophenotype, characterized by diminished gene expression in innate and adaptive immune functions such as toll-like receptor and T-cell receptor signaling pathways, was linked to unfavorable prognosis (83). Endophenotype indicators, specifically *BPGM* and *TAP 2*, were identified to enhance clinical application, predicting Mars1 (83). TAP 2 and TAP 1 constitute the TAP complex, responsible for transporting exogenous protein fragments (peptides) to the endoplasmic reticulum. The peptide is presented on the cell surface by major histocompatibility complex (MHC) class I proteins and identified by CD8+ T lymphocytes. If the peptide is detrimental, the immune system combats the intruders (87). These findings underscore the significance of personalized treatment strategies informed by molecular markers, rather than broad-spectrum medicines.

Research indicates that sepsis subphenotypes influence the effectiveness of immunomodulatory therapies (**Table 2**). A retrospective examination of gene expression in 176 septic shock patients from the VANISH clinical study, utilizing mRNA data, identified two SRS subphenotypes (SRS 1 and SRS 2) and assessed their impact on reactions to vasoactive drugs and steroids (86). The VANISH clinical trials randomly allocated patients to receive either norepinephrine or vasopressin within six hours of shock onset, thereafter administering hydrocortisone or a placebo (43). Patients displaying the immunosuppressive SRS 1 signature had a neutral response to steroid therapy, while those with the immune-active SRS 2 signature encountered worsened results (86). The restricted sample size may have negatively impacted the SRS 2 subgroup, resulting in an increased mortality rate. Numerous studies have investigated transcriptome-derived phenotypes, particularly with corticosteroid therapy. In 2020, researchers analyzed and incorporated 12 GEO and ArrayExpress datasets containing whole blood gene expression data from 1,613 adult sepsis patients (88). This research identified two sepsis subtypes (Class 1 and Class 2) by the application of deep learning methodologies and autoencoders for feature extraction. Class 1, characterized by immunosuppression, exhibited a significantly higher death rate compared to Class 2 (88). The research additionally examined the influence of sepsis subtypes on hydrocortisone response by a secondary analysis of an independent VANISH trial dataset (43, 88). The use of hydrocortisone elevated mortality rates in Class 2 patients, but not in Class 1 patients (88). This discovery underscores the necessity for comprehensive investigations into sepsis subphenotypes to enhance clinical treatment options and deepen our understanding of the complexity and treatment responsiveness of sepsis.

**Table 2:** Principal papers referenced in the text concerning the potential for phenotype-guided therapy in sepsis.

Number of Phenotypes	Names of Sepsis Phenotypes	Therapeutic approaches	Research type	Reference
2	Subclass (A and B)	Corticosteroids	Original research	Wong et al. (52)
2	SRS (1–2)	Corticosteroids	Secondary analysis	Antcliffe et al. (86)
2	Class (1-2)	Hydrocortisone	Secondary analysis	Zhang et al. (88)
2	Higher expression of BHSD1 or GLCCI1	Hydrocortisone vs placebo	Secondary analysis	Cohen et al. (89)
2	IA-P and IN-P	Hydrocortisone	Secondary analysis	Yao et al. (92)
4	Group (A, B, C and D)	Balanced crystalloids vs saline for fluid resuscitation	Secondary analysis	Bhavani et al. (115)
4	Phenotype (α, β, γ, and δ)	Eritoran (toll-like receptor 4 antagonist)	Secondary analysis	Seymour et al. (121)
4	Phenotype (α, β, γ, and δ)	Drotrecogin alfa (activated protein C)	Secondary analysis	Seymour et al. (121)
4	Phenotype (α, β, γ, and δ)	EGDT as standard care vs usual care	Secondary analysis	Seymour et al. (121)
4	Cluster (dA, dB, dC and dD)	recombinant Human thrombomodulin treatment	Secondary analysis	Kudo et al. (133)
2	Hypoinflammatory and hyperinflammatory	Drotrecogin alfa (recombinant Human Activated Protein C)	Secondary analysis	Sinha et al. (145)
2	Hypoinflammatory and hyperinflammatory	Corticosteroids	Secondary analysis	Neyton et al. (149)
4	Profile (1-4)	Different amounts of fluid resuscitation	Retrospective study	Zhang et al. (150)
5	Class (1-5)	Tailor fluid resuscitation strategy	Original research	Ma et al. (152)
4	MOF, RD, ND, and OP	Fluid balances	Retrospective study	Shald et al. (151)
2	Cluster (1-2)	Tailor fluid resuscitation strategy	Original research	Zhang et al. (109)

SRS, sepsis response signature; IA-P, immune-adaptive prevalent; IN-P, immune-innate prevalent; EGDT.

early, goal-directed therapy; MOF, multi-organ failure; RD, respiratory dysfunction; ND, neurologic dysfunction; OP, other patients.

A further work in 2021 analyzed the correlation between corticosteroid responsiveness in septic shock patients and adrenocortical gene expression (89). A thorough analysis of whole blood RNA sequencing data from 697 patients across 28 medical-surgical ICUs involved in the ADRENAL experiment revealed no correlation between adrenal cortical gene expression levels and mortality (46, 89). A logistic regression analysis demonstrated an atypical occurrence: those treated with hydrocortisone with elevated Glucocorticoid-induced transcript 1 (*GLCCI1*) gene expression levels recovered from shock more rapidly than those with increased beta-hydroxysteroid dehydrogenase 1 (*BHSD1*) gene expression (89). And a 2022 study using a classifier based on 33 gene expression patterns to classify 13 sepsis datasets into three categories (90–92). The researchers subsequently identified a minimal collection of predictive genes from differentially expressed genes (DEGs) within each subclass to accurately classify subclasses and reclassify gene expression patterns into immune-adaptive, immune-innate, and immune-coagulant subphenotypes (92). Given that the coagulant malfunction phenotype may not directly influence the response to glucocorticoid therapy, the researchers re-evaluated 117 patients from the VANISH clinical trial with septic shock, categorizing them as immune-adaptive or immune-innate, and investigated the impact of these subtypes on steroid therapy (92). The research indicated that glucocorticoids elevated death rates in the high immune adaptability cohort, supporting previous results from smaller studies and demonstrating that transcriptomically generated phenotypes could influence treatment choices (92). Patients in septic shock may exhibit varying corticosteroid responses attributable to gene expression differences. There is increased research on transcriptome-derived phenotypes and immune cell characteristics in individuals with sepsis (93). These findings assist in prognostic predictions and the assessment of treatments such as corticosteroids and vasoactive agents, which influence patients variably based on their immunological condition.

Thus, transcriptomics research has enhanced our comprehension of sepsis heterogeneity and demonstrated the potential for phenotype-based therapeutics. Precision medicine in sepsis enables the customization of therapy based on the patient’s molecular profile, enhancing diagnosis and outcomes. The discovery and clinical application of molecular classifiers are essential for enhancing therapeutic regimens and patient survival rates. Transcriptomics offers valuable insights into gene expression related to sepsis, although it does not directly reflect functional outcomes. Proteomics examines proteins, the functional outcomes of gene expression, to enhance comprehension of sepsis heterogeneity.

#### 3.1.3 Proteomics

The heterogeneity of patient reactions in sepsis complicates clinical care and impedes the development of effective, personalized therapeutics (6, 94, 95). Proteomics provides insights into the functional protein landscape in sepsis, unveiling key biomarkers associated with disease severity and outcomes (96, 97). By identifying important plasma proteins and their connections with disease severity, organ dysfunction, and patient outcomes, proteomics aids in the understanding of the mechanisms behind sepsis heterogeneity and the development of precision medicine solutions.

Recent studies have shown the effectiveness of proteomic markers in stratifying patients with sepsis. In 2022, researchers employed proteomics to differentiate between children with sepsis who developed ARDS and those who did not (97). The study identified significant markers, including S100A8, S100A12, and superoxide dismutase (SOD), in plasma that were higher in individuals with ARDS (97). S100A8/A9, a calcium-binding heterodimer predominantly located in neutrophils and monocytes, possesses pro-inflammatory and immunosuppressive characteristics and is integral to the etiology of sepsis. It is considered a potential biomarker for sepsis and its related organ damage (98). Therapeutic approaches targeting S100A8/A9 may help reduce tissue damage resulting from inflammation and enhance prognosis. The findings facilitated the creation of a proteomic signature that effectively distinguished between sepsis with and without ARDS, offering essential insights into the relevant immune systems and pinpointing potential therapeutic targets. Another large-scale study employed high-throughput mass spectrometry to examine plasma proteomes from over 1,600 sepsis patients, merging data with leukocyte transcriptomes to identify protein indicators and co-expression modules linked to sepsis severity (96). This study identified three distinct proteomic subgroups (SPC 1, SPC 2, and SPC 3) that were associated with clinical outcomes such as organ failure and death (96). For example, SPC 1 patients, who had the most severe sickness symptoms, had significantly higher mortality rates than the other subgroups (96). This classification stresses the importance of proteome profiling in identifying high-risk individuals and guiding treatment decisions based on the specifics of their sepsis phenotype.

Proteomics elucidates sepsis heterogeneity and facilitates the development of biomarkers that more accurately forecast disease progression and treatment outcomes. This establishes the foundation for individualized sepsis therapy that focuses on particular metabolic pathways. Proteomics may facilitate the identification of novel pharmacological targets, enhance patient classification, and optimize sepsis management, hence improving patient outcomes. Proteomics emphasizes functional molecules and proteins; nonetheless, it inadequately encompasses the biochemical dynamics of sepsis, which are crucial for understanding the systemic changes associated with the disease. Metabolomics bridges this gap by demonstrating the metabolic alterations associated with sepsis in a more dynamic manner.

#### 3.1.4 Metabolomics

Metabolomics examines metabolites, the end products of cellular processes, offering a snapshot of the biochemical changes during sepsis. Investigating metabolic alterations associated with sepsis phenotypes may reveal novel biomarkers for early identification, facilitate the prediction of organ failure, and enhance therapeutic strategies for specific patient subgroups, ultimately improving prognosis (99, 100). A 2015 study demonstrated the efficacy of metabolomics in the early identification of sepsis by differentiating between children requiring intensive care and those suitable for emergency room treatment (99). A combination of 14 metabolites and three protein mediators accurately indicated patients for ICU admission, highlighting the significance of metabolomics in shaping triage decisions and enhancing early intervention (99). While these findings are intriguing, they require validation in larger cohorts.

Current study has utilized metabolomics to identify organ dysfunction early in sepsis, in addition to detecting metabolic abnormalities. Lipid mediator profiling indicated that non-survivors exhibited elevated levels of inflammatory and pro-resolving mediators, correlating with severe respiratory failure and ARDS (3, 100–107). A 2021 study identified three distinct metabolic groups in patients with early sepsis and sepsis-related ARDS, each associated with different mortality rates linked to plasma lipid levels (107). Patients with high lipid levels exhibited the lowest mortality rates and a diminished risk of organ dysfunction (107). Notwithstanding these promising outcomes, the therapeutic efficacy of the notion remains uncertain, necessitating further validation. A recent study distinguished sepsis from septic shock by analyzing plasma metabolites within 48 hours of ICU admission, uncovering distinct metabolic patterns that may facilitate early diagnosis and treatment timing (108). Nonetheless, additional comprehensive validation studies and improvement of metabolite combinations remain necessary for early risk prediction.

Metabolomics has the potential to transform sepsis treatment by enhancing early detection, forecasting organ failure, and distinguishing phenotypic subgroups with varying reactions to medication. Similar to transcriptomics and proteomics, metabolomic data alone cannot adequately capture the complexity of sepsis phenotypes. Metabolomics reflects the biochemical changes occurring during sepsis, it does not account for the cellular processes and immune system dysfunction that drive disease progression. Cytomics, which focuses on the phenotypic and functional diversity of immune cells, is essential for completing the biological picture of sepsis phenotyping, providing deeper insights into immune dysregulation and aiding in the development of more personalized therapeutic strategies.

#### 3.1.5 Cytomics

Cytomics investigates the phenotypic and functional heterogeneity of cells, especially immune cells, offering real-time insights on immunological dysregulation in sepsis. Advancements in single-cell RNA sequencing (scRNA-seq) and immune cell profiling have enhanced the understanding of the pathophysiology and heterogeneity of sepsis. Specific immune cell subsets and statuses, such as MS1 cells, identified as one of the four mononuclear states, have been linked to increased mortality and organ dysfunction in sepsis patients in research, serving as potential biomarkers for sepsis prediction and therapeutic intervention (83–85). Flow cytometry and other immune profiling methodologies enhance scRNA-seq by enabling more efficient and cost-effective assessments of immune dysfunction, which may be monitored in real-time to predict patient outcomes. For example, monocyte human leukocyte antigen-DR (mHLA-DR), part of MHC-II expressed by antigen-presenting cells, is commonly measured by flow cytometry (86–88). Studies have shown that changes in the mHLA-DR expression pattern are strongly associated with a poor outcome in individuals with septic shock (89–91). These findings underscore the significance of mHLA-DR as a predictive biomarker and highlight the necessity of incorporating data from several immunological markers to obtain a holistic understanding of immune responses in sepsis. Ultimately, although each omics method provides valuable insights, the integration of these diverse datasets is essential for comprehending the complete complexity of sepsis and formulating effective, phenotype-driven therapeutics.

#### 3.1.6 Integrating multi-omics data for precision medicine

Sepsis heterogeneity is observable by the integration of epigenomic, transcriptomic, proteomic, metabolomic, and cytomic data. Each omics layer elucidates the molecular causes of sepsis, although their integration is the most potent. Researchers can identify stable biomarkers, molecular layer interactions, and dynamic changes by integrating this information (109). Transcriptomics quantifies gene expression, proteomics analyzes post-translational changes, and metabolomics examines downstream metabolic processes. These data augment our comprehension of sepsis etiology at the systemic level. Multi-omics integration revolutionizes sepsis research. High-dimensional datasets necessitate the utilization of machine learning and network-based technologies for their integration and interpretation. The integration of multi-omics enhances the classification of sepsis subphenotypes, predictions of therapy responses, and the development of the illness. Multi-omics investigations have elucidated patterns of immune dysregulation and metabolic abnormalities in sepsis, facilitating tailored therapeutic approaches (109, 110). As these technologies advance, they have the potential to revolutionize sepsis management through early detection, targeted therapy, and improved long-term outcomes.

### 3.2 Clinical indicators-based studies

Understanding the variability in sepsis patient responses is essential for improving therapeutic outcomes, and AI and ML techniques are increasingly utilized to identify distinct sepsis subphenotypes based on clinical indicators (111, 112). These approaches offer novel mechanistic pathways, therapeutic targets, and biomarkers essential for developing sepsis-specific phenotype-driven therapies. For instance, unsupervised machine learning approaches such as cluster analysis allow for the classification of sepsis patients into subphenotypes without prior assumptions, utilizing clinical data such as vital signs, white blood cell counts, and biochemical markers (111, 113, 114). A 2022 study identified and validated several novel sepsis subphenotypes based on changes in vital signs over time (115). This multicenter retrospective study assessed vital signs, including body temperature, heart rate, respiratory rate, and blood pressure, in 20,729 hospitalized patients with suspected infections within the initial eight hours post-admission (115). Four sepsis subphenotypes (Groups A, B, C, and D) exhibited variations in clinical manifestations, laboratory findings, organ impairment, and prognosis (115). The secondary analysis of the SMART trial compared balanced crystalloids to normal saline in critically ill patients and demonstrated that subphenotypes influenced mortality outcomes (115, 116). Balanced crystalloids decreased mortality in Group D relative to normal saline (115). The observations indicate that sepsis subphenotypes characterized by easily obtainable vital signs may exhibit varied responses to pharmacological interventions, potentially impacting future clinical trials. Phenotype-driven therapeutic approaches customize interventions and enhance patient outcomes. Notwithstanding these advancements, the application of AI in sepsis diagnosis and treatment remains impeded by challenges such as subgroup selection and biases in training datasets (117–119). AI and ML can enhance sepsis classification and identify individualized patient response patterns, facilitating precision therapy. These approaches can revolutionize sepsis therapy by facilitating earlier, more precise actions that improve patient outcomes when integrated with genetic data.

#### 3.2.1 Diverse clinical phenotypes

Researchers have progressively employed various omics data (**Figure 3**) in conjunction with standard clinical and laboratory test data—such as white blood cell counts and biochemical markers—utilizing sophisticated big data algorithms and artificial intelligence methods to categorize sepsis phenotypes (120–123). These projects have produced substantial insights into the diversity of sepsis and its clinical implications. A 2015 study utilizing self-organizing maps (SOMs) and K-means clustering found four clinical phenotypes among 2,533 individuals with severe sepsis or septic shock, each with unique organ failure patterns and fatality rates (120). A 2020 study utilizing SOM and cluster analysis on the Medicine Information Database III (MIME-III) identified four distinct sepsis subtypes, each defined by specific organ failures, underscoring the necessity for personalized treatment strategies (124). A notable study published in 2019 using consensus K-means clustering to assess sepsis patients (n=20,189) from 12 medical institutions and three randomized clinical trials (n=4,737) (121). Within six hours of admission, 29 clinical and laboratory data points from electronic health records were employed to identify four distinct phenotypes (α, β, γ, and δ), each defined by specific clinical characteristics and associated mortality risks (121). The research examined 43,086 cases, involving 31,160 unique people, to evaluate phenotypic repeatability. Simulations of three randomized controlled trials demonstrated that altering the distribution of α and δ traits greatly affected trial outcomes (44, 121, 125, 126). This study highlights the significance of accounting for phenotypic dispersion in clinical trial design, as it can affect the efficacy of treatment interventions (121, 127, 128). This study underscores the need of identifying appropriate phenotypic classifications and administering prompt therapeutic interventions, as drugs may prove ineffective or harmful if not congruent with specific patient morphologies.

Nonetheless, machine learning methodologies exhibit a deficiency in transparency, and phenotypic consistency varies across studies. Subphenotypes are typically identified using cross-sectional clinical and biochemical data, which constrains our understanding of their temporal dynamics. Recent studies have examined dynamic sepsis subtyping according to the temporal progression of the disease (129–131). A 2022 study analyzed 642 patients across 20 hospitals, used 23 baseline clinical markers, and identified clustering through heat maps at 0, 6, 24, and 72 hours (129). Five organ failure symptoms (Types L1, L2, M, H1, and H2) exhibiting varied patterns and durations were validated in an independent sample of 381 individuals from 11 institutions (129). The two high-risk phenotypes, H1 and H2, had comparable mortality rates; however, there were significant discrepancies in baseline disease severity scores, clinical characteristics (including age and organ failure patterns), and plasma marker levels (129). Another study employed 72-hour sequential organ failure assessment (SOFA) score trajectories for sepsis patients, utilizing dynamic temporal warping (DTW) and hierarchical agglomerative clustering (HAC) to delineate subgroups based on trajectory data (130). The researchers constructed a random forest model to forecast subtype affiliation at 6 and 24 hours post-ICU admission (130). The study comprised 4,678 sepsis patients in the development cohort and 3,665, 12,282, and 4,804 patients in the validation cohorts. Four groups were identified: rapidly deteriorating, gradually declining, rapidly enhancing, and gradually improving. Distinct baselines, organ dysfunction, and clinical outcomes were present for each category. Although the rapidly deteriorating subtype exhibited a lower SOFA score at ICU admission (mean: 4.5) compared to the rapidly improving subtype (mortality rate: 5.5%, mean SOFA: 5.5), it nonetheless had the highest in-hospital mortality rate at 28.3% (130). This research identified four unique sepsis subphenotypes, each exhibiting distinct natural histories, illustrating host-pathogen interactions throughout standard treatment. Comprehending these dynamic trajectories is essential for formulating and forecasting clinical trial interventions, as it uncovers discrepancies in natural history and treatment effectiveness. Other studies employed population-based trajectory modeling to classify sepsis according to variations in body temperature, uncovering groupings with markedly distinct inflammatory profiles and fatality rates (131, 132). These strategies enhance our comprehension of sepsis progression and facilitate the development of more tailored therapeutic choices.

Alongside standard clinical indicators, phenotypic assessments encompass coagulation profiles and hemodynamic evaluations. K-means clustering was employed in machine learning including 3,694 patients from three Japanese multicenter studies conducted in 2021 (133). The research examined the reactions of different phenotypes to recombinant human thrombomodulin (rhTM) injections and the subsequent clinical outcomes (133). Four distinct coagulation phenotypes were identified: dA, dB, dC, and dD. In the dA phenotypic group, rhTM therapy decreased 28-day and in-hospital mortality, although the absence of standardization or randomization (133). These findings highlight the therapeutic potential of phenotype-driven therapies and the importance of establishing successful therapy windows. Current research faces issues in repeatability and transparency in machine learning approaches. However, collected evidence suggests that phenotype-based interventions may have a considerable impact on sepsis therapy (134–137). Integrating multi-omics data with dynamic clinical signs, such as alterations in inflammatory markers or hemodynamic parameters, is expected to enhance phenotypic classification. Longitudinal tracking of clinical changes enables researchers to associate genetic findings with disease progression and get a deeper understanding of sepsis features. These advancements facilitate the identification of novel biomarkers and therapeutic targets for sepsis subphenotypes, enhancing patient outcomes and customized treatment.

#### 3.2.2 Hyperinflammatory and hypoinflammatory subphenotypes

Sepsis, akin to other intricate diseases, exhibits heterogeneity, complicating treatment and diminishing the effectiveness of various research trials (94, 138). Distinguishing distinct biological phenotypes that may exhibit varied responses to therapies is essential for enhancing therapeutic outcomes (129, 139–144). Two principal subphenotypes in sepsis—hyperinflammatory and hypoinflammatory—have shown significant in forecasting disease progression, treatment responses, and patient outcomes (109, 145–147).

These two inflammatory types were accurately identified in numerous sepsis cohorts by latent class analysis (LCA) of clinical and biomarker data (145). The hyperinflammatory phenotype is characterized by elevated proinflammatory cytokines, utilization of vasoactive medications, heightened risk of bacteremia, and increased death rate (145). The hypoinflammatory phenotype is characterized by diminished inflammatory markers, a lower risk of bacteremia, and an improved prognosis (145). Clinical and treatment responses differ by phenotype, suggesting that phenotype-specific strategies may enhance patient outcomes. The secondary analysis of the PROWESS SHOCK trial (N=1680) assessing recombinant human activated protein C (drotrecogin alfa) in septic shock revealed that hyperinflammatory patients exhibited superior survival rates compared to hypoinflammatory patients, who demonstrated diminished survival rates (40, 145). No treatment interactions were identified in the secondary analysis of the VASST trial, which compared norepinephrine to early antidiuretic hormone therapy in cases of septic shock (145, 148). Nevertheless, distinctions consistent with other cohort studies emphasize the significance of phenotype in therapeutic classification.

Further investigation has examined the impact of these variables on mortality associated with sepsis-related ARDS (146). Hypoinflammatory patients predominantly succumbed to respiratory failure, while hyperinflammatory patients primarily perished from circulatory failure (146). The data indicate that addressing individual inflammatory profiles may enhance treatment customization and effectiveness. Genomic and microbiomic data suggest that hyperinflammatory individuals have elevated bacterial numbers and immunological activity, impacting metabolic and T cell response genes (149). Researchers employed this methodology on VANISH trial participants with transcriptome data (N=117), classifying them as hypoinflammatory or hyperinflammatory and evaluating phenotype-specific treatment benefits by logistic regression models (43, 149). Steroid medication influenced various phenotypes differently, with hypoinflammatory patients perhaps encountering adverse outcomes (149). The diverse sepsis phenotypes underscore the necessity of tailored treatment according to clinical severity and genetic factors. Phenotype-based predictive enrichment in clinical trials is advantageous as hyperinflammatory and hypoinflammatory phenotypes respond differentially to treatment.

Classifying patients based on their inflammatory profiles may aid in identifying individuals who benefit most from various medications, thereby improving the efficacy and safety of sepsis treatment. Defining and characterizing sepsis hyperinflammatory and hypoinflammatory subphenotypes clarifies clinical heterogeneity and facilitates phenotype-guided therapy. Examining biomarkers and therapeutic targets related to these traits could improve patient outcomes, inform clinical decision-making, and increase the effectiveness of sepsis treatment.

#### 3.2.3 Fluid resuscitation and recovery

Effective fluid management is essential in sepsis and septic shock to enhance hemodynamic stability and organ perfusion (28). A phenotype-driven strategy for fluid therapy is essential, since both excessive and inadequate fluid resuscitation can adversely affect patient outcomes. Research indicates that fluid resuscitation affects mortality risk and recovery across many sepsis subtypes (150, 151). A 2018 Latent Profile Analysis of 14,993 sepsis patients showed four categories regarding fluid balance and mortality risk (150). The results indicated that responses to fluid resuscitation varied markedly among different morphologies. Subtype 2 got a lesser volume of fluid compared to subtype 3, which necessitated a greater volume within 24 hours. Notably, augmenting fluid administration during the initial 48 hours elevated mortality in subtype 4, while concurrently reducing mortality in subtype 3, which exhibited hallmarks of circulatory shock, including diminished mean arterial pressure and heightened vasopressor requirements (150). These findings underscore the necessity of tailoring fluid resuscitation to the individual characteristics of each patient.

A dynamic treatment regime (DTR) model enhanced fluid resuscitation for septic shock in 2021. More than 1,400 patients were assessed to delineate five phenotypes, ranging from the most severely ill (Phenotype 2) to the healthiest (Phenotype 5) (152). Early, vigorous fluid resuscitation succeeded by a de-resuscitation period to mitigate fluid excess was determined to be the optimal fluid management strategy. Phenotype-specific fluid quantities and norepinephrine dosages enhanced mortality outcomes, with certain patients gaining advantages from early administration, while others, particularly the critically ill, benefitted from delayed treatment (152). This study indicates that treatment for sepsis should be tailored to its varied phenotypes. A 2022 study investigated fluid balance in patients with clinical sepsis to validate these findings (151). Patients were classified into categories of multiple organ failure (MOF), respiratory dysfunction (RD), nervous system dysfunction (ND), and others (151). The data indicated considerable phenotypic differences in fluid balance and mortality. MOF exhibited the highest mortality rate at 48.4%, whilst OP demonstrated the lowest at 13.7% (151). At different time intervals post-therapy, MOF and ND patients exhibited the highest fluid volumes balances, indicating that fluid management must be customized for each phenotype.

A recent study proposed utilizing transcriptome data to guide fluid management in septic shock patients through a ‘benefit score’ (109). A comprehensive review of multi-omics data from 494 septic shock patients revealed distinct subgroups with varying responses to fluid therapy strategies and established a “benefit score” derived from transcriptomic data and a proteomic signature to assist clinicians in customizing fluid management approaches (109). This study employs a systems biology approach utilizing multi-omics data to enhance our comprehension of sepsis heterogeneity and to offer novel strategies for personalized treatment. These findings are valuable; nevertheless, prospective validation, rapid detection methods, and cost-effectiveness analysis are necessary prior to their implementation in clinical practice.

These studies underscore the importance of accurate fluid management in sepsis treatment, along with comprehending the molecular and clinical diversity of sepsis phenotypes. Identifying and targeting phenotype-specific fluid resuscitation methods enables the optimization of treatment, diminishes complications, and ultimately enhances patient outcomes in sepsis and septic shock.

## 4 Future directions for sepsis phenotyping

### 4.1 Advancing phenotype-guided therapies

Investigations into sepsis subphenotypes have yielded essential understanding of the disease’s heterogeneity and shown the potential for phenotype-directed therapeutic strategies. The results underscore the need for additional validation and enhancement in converting subphenotypic insights into effective treatment strategies that enhance patient outcomes. A deeper comprehension of sepsis heterogeneity is essential for developing phenotype-specific treatments. Current subphenotypic research are significant, but they are limited by a lack of standardized validation across diverse demographics and clinical circumstances. Numerous studies depend on retrospective or cohort data (**Table 3**); nevertheless, prospective research is crucial for creating effective, real-time phenotyping instruments that can immediately impact treatment decision-making (153–155). Clinical trials have shown that subphenotypes are associated with different treatment responses, emphasizing the necessity of screening these phenotypes ahead of time to optimize therapy and enhance prognosis. Illustrations from various domains exemplify the pragmatic efficacy of phenotype-guided therapy. In a chronic pain management study, 60 patients were randomly divided into two groups: one receiving opioid medicine guided by CYP2D6, μ-opioid receptor (*OPRM1*), and catechol-O-methyltransferase (*COMT*) genotyping, and the other receiving standard care (156). The genotype-guided group experienced significantly better pain relief and quality of life compared to the control group. This case underscores the significance of integrating genetic and phenotypic data to enhance therapeutic outcomes. Similar strategies could be adapted for sepsis management, emphasizing the need for prospective studies to evaluate their applicability.

**Table 3:** Comparison of multi-omics and clinical indicators in sepsis phenotyping.

Category	Multi-omics-Based Approach	Clinical Indicator-Based Approach
Core Objective	➢ Elucidate sepsis heterogeneity; enable phenotype-driven therapies.	➢ Rapid phenotype assessment to support clinical decisions.
Advantages	• Reveals biological mechanisms and therapeutic targets. • Supports precise stratification and personalized treatment. • Enhances reproducibility and robustness of research outcomes.	• Widely accessible, simple to implement. • Enables real-time monitoring and adaptive treatments. • Suited for resource-constrained settings.
Limitations	♦ High costs and lengthy development cycles. ♦ Reliance on advanced data integration technologies.	♦ Variability in patient populations and sampling timepoints. ♦ Limited generalizability of findings to diverse cohorts.
Key Insights	▪ Phenotype-driven identification of novel biomarkers and therapeutic targets. ▪ Integration of machine learning for dynamic classification.	▪ Improved data standardization and real-time monitoring platforms.. ▪ Optimized clinical workflows for phenotype-based strategies.
Future Directions	➢ Refine individualized therapies based on phenotypic diversity. ➢ Establish multicenter collaborations for data sharing and validation.	➢ Enhance synergy with multi-omics approaches. ➢ Develop standardized tools for clinical phenotype evaluation.

### 4.2 Challenges in sepsis phenotyping

Sepsis phenotyping has several challenges, including data heterogeneity, validation difficulties, and the integration of emerging techniques such as ML and AI. The variability in patient demographics, clinical environments, and sampling methods restricts the generalizability and reliability of phenotyping studies (157, 158). Establishing uniform terminology such as “phenotype,” “subphenotype,” and “subgroup” is essential for enhancing clarity and collaboration. Standardized methods, precise nomenclature, and cohesive research methodologies are essential to resolve overlaps among subphenotypes and enhance classification systems for precision medicine.

A notable challenge is the limited accessibility of contemporary multi-omics technologies, attributed to elevated costs and technical intricacies, especially in resource-limited settings (1). Phenotypic models should be tested across diverse populations to guarantee their global applicability and fairness in sepsis research. Interdisciplinary collaboration integrating clinical, biological, and computational expertise is crucial for synthesizing diverse datasets and improving phenotype-guided methodologies. International efforts to standardize procedures and disseminate data can accelerate discoveries and facilitate access to advanced methodologies.

Emerging tools, including machine learning and artificial intelligence, can identify underlying diversity in treatment responses, even within large studies that yield neutral results (130, 159–161). Despite the potential of machine learning and artificial intelligence to find variability in treatment responses and uncover novel biomarkers, obstacles such as insufficient transparency, scalability issues, and challenges in integrating into clinical workflows hinder their widespread adoption (162, 163). Developing economical, interpretable AI models and scalable platforms is essential for practical implementation. Moreover, robust ethical frameworks for data protection and governance are essential for the proper utilization of patient data. Surmounting these obstacles will advance sepsis phenotyping, facilitating more tailored therapies and improved patient outcomes.

### 4.3 Expanding the scope of phenotypic research

#### 4.3.1 Cross-organ system investigations

A comprehensive understanding of sepsis requires extending beyond blood-based evaluations to incorporate cross-organ system analyses that elucidate the complex interactions influencing disease progression (164). Although omics technologies provide significant molecular insights, the gap between these findings and clinical results highlights the necessity for more thorough phenotypic evaluations. Sepsis often impacts many organ systems, resulting in differing levels of dysfunction; therefore, it is essential to investigate gene expression, protein function, and metabolite regulation across tissues (13). Broadening phenotypic studies to encompass organ-specific abnormalities, particularly in the lungs, liver, and kidneys, will enhance our comprehension of sepsis etiology and facilitate the creation of therapies aimed at organ-specific harm (20, 165, 166). Future study ought to integrate multi-omics methodologies with cross-organ studies to develop a whole comprehension of sepsis and propel advancements in detection and treatment.

#### 4.3.2 The temporal dynamics of phenotypes

**Figure 4:**
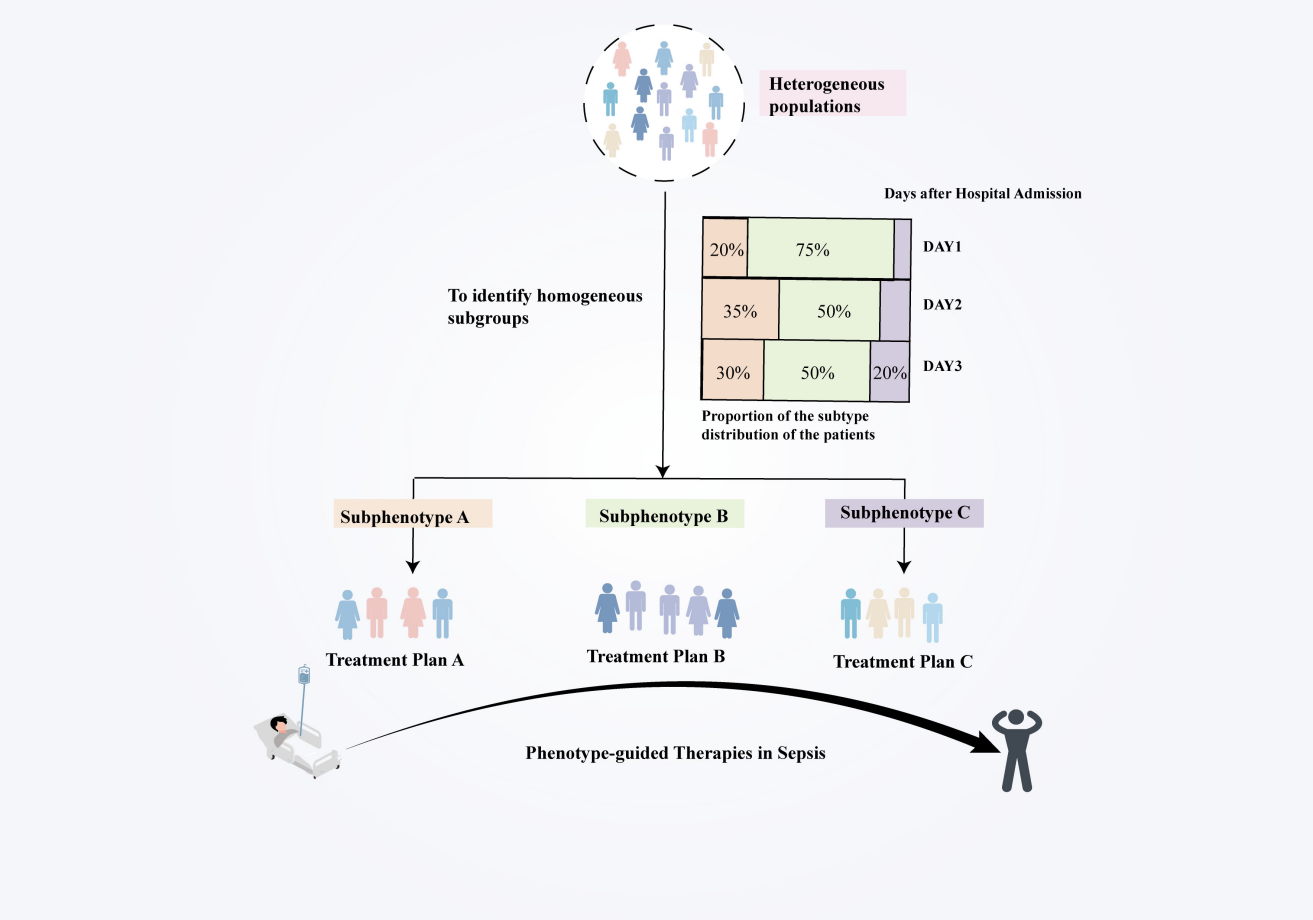
Dynamic subtyping and real-time decision-making address sepsis heterogeneity by combining clinical, biomarker, and molecular data to identify more homogeneous subpopulations. This methodology promotes phenotype-driven medicines, facilitates the discovery of new markers and targets through various treatment responses, and improves patient outcomes by providing accurate pharmaceuticals to the appropriate individuals at the right time.

The evolving characteristics of sepsis phenotypes present both challenges and opportunities for precision treatment (167). Determining the optimal intervention window and tracking phenotypic changes is critical for tailoring therapy to individual patients and improving long-term outcomes (**Figure 4**). Nevertheless, the majority of contemporary research employs single time-point cross-sectional data, which constrains their ability to differentiate between ephemeral chemical states and persistent phenotypic characteristics. For example, phenotypes identified in early hyperinflammatory stages may shift to immunosuppressive states during later stages of sepsis progression (147). Without longitudinal sampling, it is challenging to distinguish transient changes from stable molecular characteristics, potentially leading to misclassification. This limitation raises the concern that current subtyping studies may reflect transient stages of sepsis progression rather than stable, distinct patient subtypes. longitudinal sampling, coupled with multi-omics integration, can surmount these limitations by monitoring molecular and clinical markers over time. This method facilitates the discovery of stable phenotypes, improves categorization systems, and directs the development of stage-specific therapies. Emphasizing dynamic studies will allow researchers to customize treatment strategies to the evolving characteristics of sepsis, enhancing patient outcomes and advancing the field of precision medicine.

## 5 Conclusions

Understanding the heterogeneity of sepsis is pivotal for advancing phenotype-based therapeutic strategies and improving patient outcomes. This review synthesizes advances in clustering algorithms, multi-omics technologies, and artificial intelligence, providing a comprehensive framework for identifying distinct sepsis subtypes with varied treatment responses. These insights lay the groundwork for discovering novel biomarkers and therapeutic targets, underscoring the transformative potential of precision medicine to tailor treatments based on phenotypic diversity. However, challenges such as phenotyping standardization, dynamic data integration, and clinical translation must be addressed through interdisciplinary collaboration and innovative research. By bridging these gaps, future efforts can fully implement phenotype-driven therapies, transforming sepsis management, enhancing survival rates, and establishing a new paradigm for precision medicine in complex diseases.
